# Sample Size Calculations for Population Size Estimation Studies Using Multiplier Methods With Respondent-Driven Sampling Surveys

**DOI:** 10.2196/publichealth.7909

**Published:** 2017-09-14

**Authors:** Elizabeth Fearon, Sungai T Chabata, Jennifer A Thompson, Frances M Cowan, James R Hargreaves

**Affiliations:** ^1^ Department of Social and Environmental Health Research London School of Hygiene and Tropical Medicine London United Kingdom; ^2^ Department of Infectious Disease Epidemiology London School of Hygiene and Tropical Medicine London United Kingdom; ^3^ Centre for Sexual Health and HIV/AIDS Research Harare Zimbabwe; ^4^ Department of International Public Health Liverpool School of Tropical Medicine Liverpool United Kingdom

**Keywords:** population surveillance, sample size, sampling studies, surveys and questionnaires, research design, data collection, sex workers, HIV

## Abstract

**Background:**

While guidance exists for obtaining population size estimates using multiplier methods with respondent-driven sampling surveys, we lack specific guidance for making sample size decisions.

**Objective:**

To guide the design of multiplier method population size estimation studies using respondent-driven sampling surveys to reduce the random error around the estimate obtained.

**Methods:**

The population size estimate is obtained by dividing the number of individuals receiving a service or the number of unique objects distributed (*M*) by the proportion of individuals in a representative survey who report receipt of the service or object (*P*). We have developed an approach to sample size calculation, interpreting methods to estimate the variance around estimates obtained using multiplier methods in conjunction with research into design effects and respondent-driven sampling. We describe an application to estimate the number of female sex workers in Harare, Zimbabwe.

**Results:**

There is high variance in estimates. Random error around the size estimate reflects uncertainty from *M* and *P*, particularly when the estimate of *P* in the respondent-driven sampling survey is low. As expected, sample size requirements are higher when the design effect of the survey is assumed to be greater.

**Conclusions:**

We suggest a method for investigating the effects of sample size on the precision of a population size estimate obtained using multipler methods and respondent-driven sampling. Uncertainty in the size estimate is high, particularly when *P* is small, so balancing against other potential sources of bias, we advise researchers to consider longer service attendance reference periods and to distribute more unique objects, which is likely to result in a higher estimate of *P* in the respondent-driven sampling survey.

## Introduction

Population size estimates (PSE) for those most at risk for human immunodeficiency virus infection are crucial to make epidemic projections, allocate funding, and monitor coverage of prevention and care programs [1,2]. However, these populations are frequently stigmatized and criminalized and it is often not feasible or practical to conduct a census. One approach to obtaining a PSE is to use multiplier methods, including the service multiplier method (SMM) and the unique object multiplier method (UOM). The former uses 2 sources of data: (1) a count of program attendance or receipt of a service targeted to the population in question, and (2) a representative survey of the population in which uptake of service can be determined. The latter is the same, except the count is of the number of recognizable objects distributed to a population in advance of a survey. Obtaining a random sample of a population lacking a sampling frame is challenging, but there has been guidance published on adapting one of the methods commonly in use, respondent-driven sampling (RDS) [3], for use with the service multiplier method [4]. 

While there has been research into sample size requirements for RDS surveys [5-7], we lack guidance applied to sample size requirements when used to obtain a PSE with a multiplier method. Here, we report our approach in the context of preparing a protocol to estimate the number of female sex workers (FSW) in Harare, Zimbabwe using the SMM implemented with an RDS survey.

## Methods

### Overview

We briefly outline multiplier method size estimation, the approach to estimating uncertainty in the resulting population size estimates, and integrate this with advice on design effects and sample size requirements for RDS surveys.

### Multiplier Method Population Size Estimation

**Figure 1:**
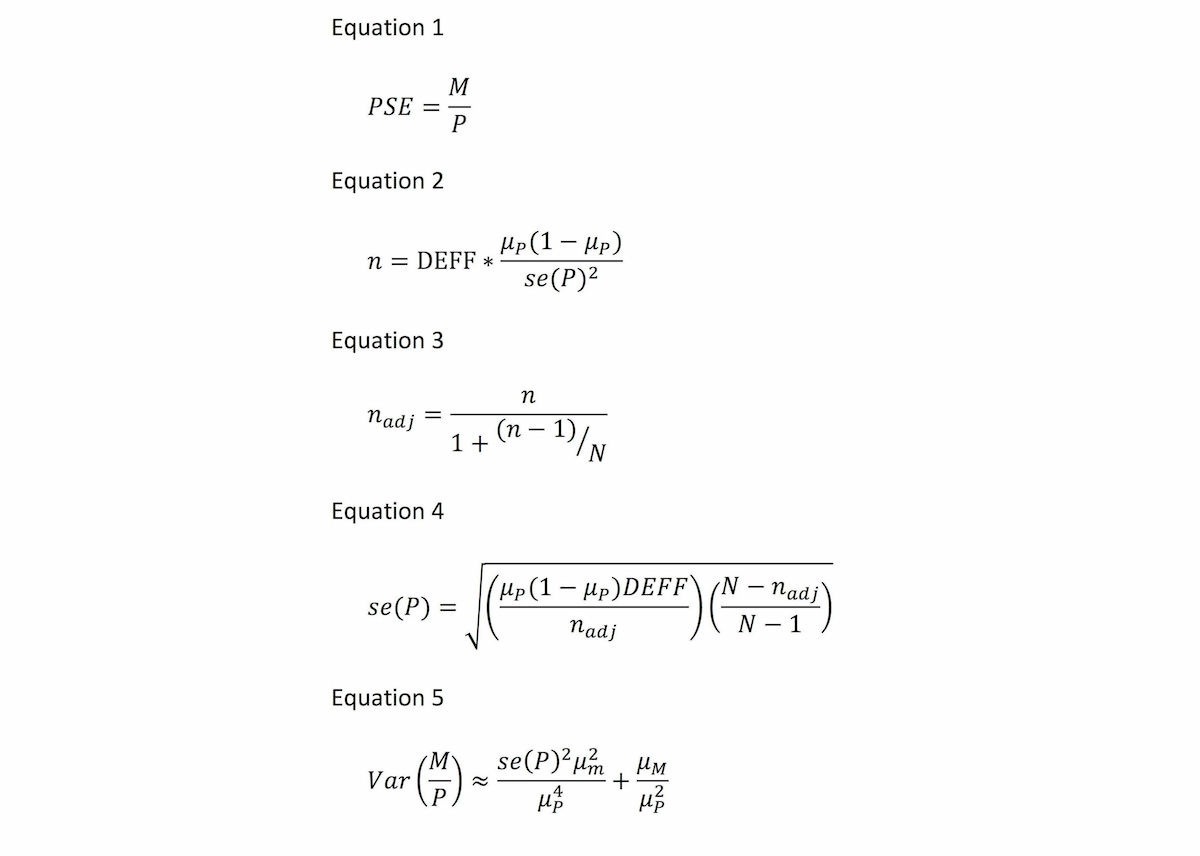
Equations for estimating population size, study sample size, and variance of the population size estimate.

Multiplier methods use 2 sources of data to estimate population size as described above: (1) a count of unique individuals from the target population receiving a service or unique objects distributed among this population, *M*, and (2) a representative estimate of the proportion of the target population in receipt of the service or object, *P*. The count is divided by the proportion as in Equation 1 (Figure 1) to obtain the population size estimate.

Johnston et al. [4] suggest using the Delta method to estimate the variance of the PSE, which combines variance in *P* and variance in *M*. We assume that *M*, as a count of target population individuals on a roster or unique objects distributed to the target population, follows a Poisson distribution for which the mean and variance are equal to µM [8].  The variance of *P* depends on the sample size of the RDS survey.

### Sample Size Calculations

RDS is a structured, peer-referral recruitment method assuming a model for estimating each participant’s probability of inclusion; thus, allowing weighting of responses to be used to approximate a random sample [9]. Existing guidance for estimating proportions from a RDS survey suggests that the sample size required for a simple random sample must be multiplied by a design effect (DEFF) to account for the RDS design [10]. Empirical reviews of RDS surveys have found most DEFFs to lie between 2 and 4, though some studies have found higher DEFFs [5-7,11]. The sample size for the RDS survey used to estimate *P* can be calculated as Equation 2 (Figure 1) given that *n* is sample size, *µ*_P_ is the estimate for the proportion we wish to estimate, and *se(P)* is the standard error of *P*. Recognizing that PSE are often required for small sites, we additionally suggest using a sample size *n*_adj_ that has been corrected for an estimated finite population as Equation 3 (Figure 1), where *N* is the estimated population size.

Rearranging Equation 2, and using *n*_adj_ as obtained in Equation 3, *se(P)* as corrected for finite population size can be calculated as Equation 4 (Figure 1), and the effect on the variance of the PSE can be obtained by inserting *se(P)* into Equation 5 (Figure 1). The 95% confidence interval (CI) around the PSE can then be obtained by taking the square root of *var(M/P),* multiplying by 1.96 (assuming an approximately normal distribution) and subtracting/adding to the PSE.

We examined the relationship between sample size, *P*, and the width of the 95% CI obtained for a population size estimate of 15,000, fixing this estimate so that *M* varied with *P*.

### Application to Estimating the Number of Female Sex Workers in Harare

To estimate the number of FSW in Harare, we planned a RDS survey of FSW aged 18 and older who had resided in the city for at least the previous 6 months. For service data, we planned to use Sisters with a Voice clinic attendance records. FSW attending this clinic, which provides sexual and reproductive health services for self-identified FSW, are given unique identification numbers and their visits recorded and dated (described further elsewhere [12]). For *M*, we planned to record the number of unique women attending in the 6 months prior to the survey.

To identify a reasonable estimated FSW population size for sample size calculation, we used previous estimates from a systematic review of FSW prevalence among 15- to 49-year-old women in sites from sub-Saharan Africa (.07%–4.3%) and multiplied them by the number of women of this age in Harare [13]. The 2012 Zimbabwe census estimates that 30.2% of the population of Harare is female aged 15 to 49, and that the total population of Harare is 2,123,132 [14], giving a FSW population size in Harare of 4488 to 27,572, with a plausible midrange estimate of 15,000, or 2.3%, of the adult female population.

We examined the number of sex workers who visited the program for different reference periods up to April 23, 2015 to generate likely values for  *M* and  *P* given an assumed PSE of 15,000. We then examined the impact of reference period on sample size requirements assuming these values of  *M* and  *P*. Finally, we investigated the effect of DEFF on the width of the 95% CIs around the PSE for different sample sizes of the RDS survey. We developed a Web-based tool to implement the methods described here [15].

## Results

### Relationships Between RDS Survey Sample Size, P, M, and Width of the 95% Confidence Intervals

**Figure 2:**
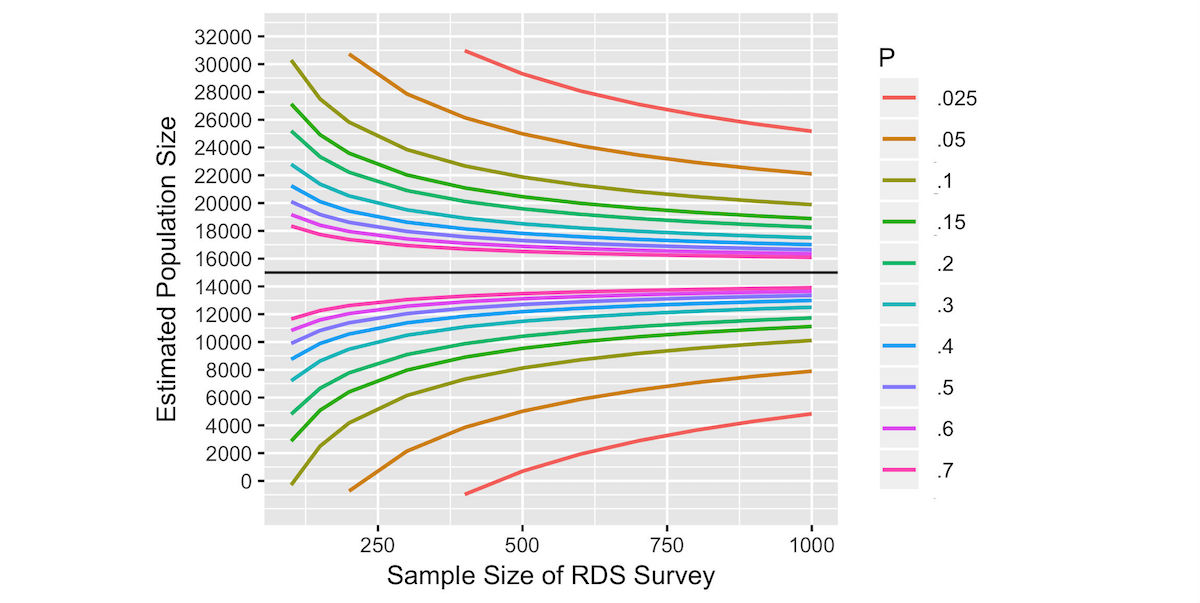
Sample size and width of 95% confidence interval around a fixed population size estimate of 15,000 for different values of *P* and *M*, assuming a design effect of 3.

For all values of *P* and *M,* increasing the RDS survey sample size decreases the width of the CI around the PSE, Figure 2. The precision of the PSE also varies by the values of *P* and *M*, such that much larger sample sizes would be required to estimate the PSE with the same level of precision if *P* is small rather than large (and correspondingly, *M* is small rather than large).

In Figure 2, values of *M* are varied with *P* so that *M* / *P* is always equal to 15,000. For instance if *P*=.05, *M*=750, or if *P*=.4, *M*=6000.

### Application to Planning a Population Size Estimation Study

**Figure 3:**
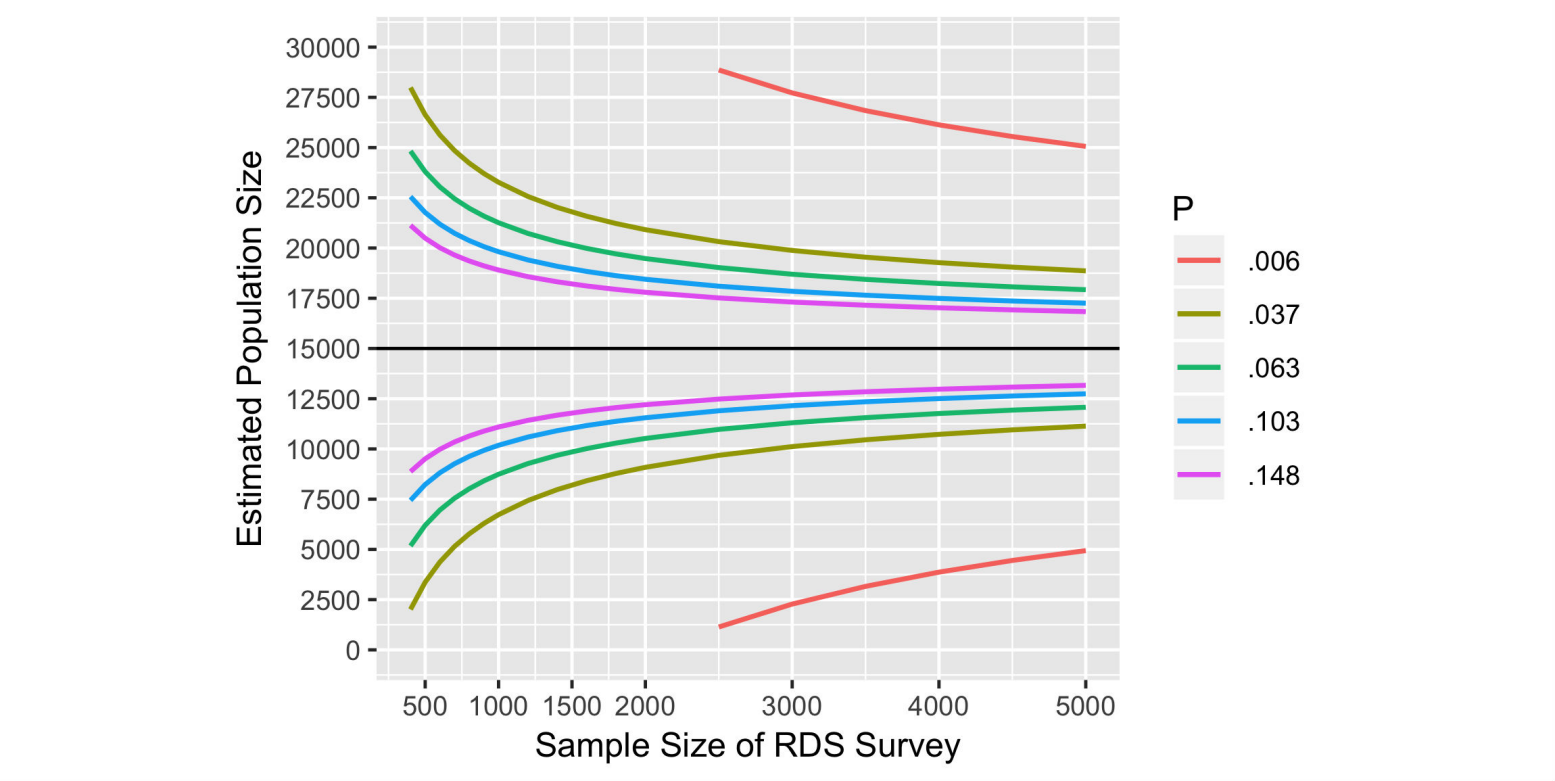
Effect of reference period (variations in *P*), width of the 95% confidence interval around the population size estimate and sample size required for estimating the number of female sex workers in Harare.

**Figure 4:**
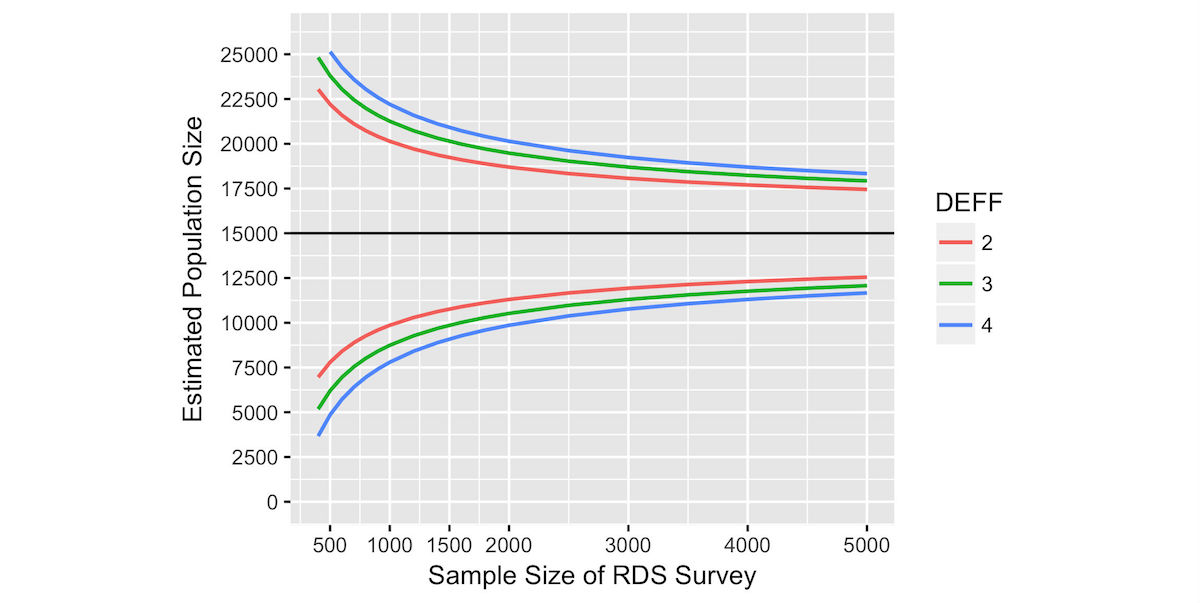
Sample size and width of 95% confidence intervals around a population size estimate of 15,000 female sex workers in Harare, for assumed reference period of 6 months and design effects (DEFF) of 2, 3, and 4.

For our Harare example, we were able to review earlier service attendance data to see how the value of *M* might depend on the reference period chosen. The value of *M* in turn affects the sample size required via the impact on *P*, as shown in Table 1 and Figure 3, which assume a population of 15,000 FSW in Harare. Depending on whether we chose a period of 1 or 24 months, we might be estimating a proportion of .006 or a proportion of .148. For a given sample size, the width of the 95% CI will increase if the reference period is shorter and *P* is smaller. Higher DEFFs increase the uncertainty around the PSE, Figure 4.

We used previous service attendance data to observe how *M* varied by reference period, and therefore to predict how our estimate of *P*, the proportion of women attending, might vary by the reference period we chose, see Table 1. Figure 3 shows the relationship between these values of *P* with the width of the 95% CI’s around the PSE for different sample sizes.

Based on changes in the width of the estimated 95% CIs with increasing sample size (Figure 3) and on choosing a reference period that would both reduce the likelihood of recall bias while preventing *P* from being too low, we chose a sample size of 1500 FSW for the RDS survey and a reference period for Sisters service attendance of 6 months, for which we estimated *P* would be approximately .06.

**Table 1:** Number of female sex workers attending the Sisters program and effect on *P* given the total female sex worker population = 15,000 in Harare.

Reference period to April 23, 2015	Number of unique female sex workers attending, M	Estimated *P*, assuming population = 15,000
**1 month**	85	.006
**3 months**	560	.037
**6 months**	952	.063
**12 months**	1542	.103
**24 months**	2227	.148

## Discussion

### Summary and Discussion of Findings

We have applied current guidance on RDS and multiplier methods to propose an approach to planning population size estimation studies and determining sample size. We have given an example using the SMM, similar principles of which can be applied to the UOM.

Even for large sample sizes, 95% CIs around the PSE are wide. The uncertainty around the PSE is more sensitive to the uncertainty in *P* than in *M*, which is evident from the formula for *var(M/P)*. Researchers cannot choose a value of *P*, but they can encourage it to be higher by encouraging *M* to be higher. Concerned only with random error, it would improve the precision of the PSE to choose a longer reference period, and thus likely obtain a larger *P* in the case of the SMM, or to distribute a greater number of unique objects for the UOM. However, for the SMM this approach needs to be balanced against the potential for recall bias on estimation of *P*. It is also possible that the relationship between *M* and the reference period will differ across service types and according to whether individuals visit frequently or sporadically, and that bias in *M* might vary by reference period. If there are errors in unique identification of individuals in the service data, a longer reference period could lead to a higher likelihood of duplicate identification numbers, which would bias the PSE. For the UOM, care is needed to ensure that more objects distributed did not increase the likelihood of dependence between methods of distribution and RDS survey recruitment, a key source of potential bias.

We used DEFFs of 2 to 4 in our sample size calculations, but it is possible that a higher value would be more appropriate. Previous research has found that high levels of homophily (similarity) between recruiters and recruitees in RDS surveys is associated with higher DEFFs [7]. In SMM studies, the RDS survey is intended to measure program attendance, a characteristic that is likely to exhibit high homophily as it is a route by which participants might know and recruit each other. High homophily is also likely when the same social networks are used to distribute unique objects and to later recruit individuals to a RDS survey. Higher DEFFs might therefore be required, though in a previous population size estimation study of 9 communities in Zimbabwe, we found evidence of high homophily by program attendance for some sites but not all [8].

RDS surveys must have sufficient recruitment waves in order to reach stable estimates. There should also be sufficient numbers of seed participants to reflect diversity of the target population [16], concerns that need to be considered alongside the total sample size [17].

### Recommendations

This short paper considers random error around size estimates and does not discuss a consideration of bias resulting from unmet assumptions of both the multiplier and RDS methods, which we consider elsewhere [8]. We agree with advice that researchers should use more than one multiplier and more than one method of estimating population size [18,19]. However, justification for sample size is often not given. Based on our findings, we strongly recommend conducting sample size calculations for estimating population size and considering the relationship between reference period or number of objects distributed and *P* for potential impact on uncertainty.

## Abbreviations

CI: confidence interval
DEFF: design effect
FSW: female sex workers
PSE: population size estimates
RDS: respondent-driven sampling
SMM: service multiplier method
UOM: unique object multiplier method
